# Health and economic burden estimates of snakebite management upon health facilities in three regions of southern Burkina Faso

**DOI:** 10.1371/journal.pntd.0009464

**Published:** 2021-06-21

**Authors:** Sayem Ahmed, Guibehi B. Koudou, Maïwenn Bagot, François Drabo, Windtaré R. Bougma, Caisey Pulford, Moses Bockarie, Robert A. Harrison

**Affiliations:** 1 The Centre for Snakebite Research & Interventions, Department of Tropical Disease Biology, Liverpool School of Tropical Medicine, Liverpool, United Kingdom; 2 The Centre for Neglected Tropical Diseases, Department of Tropical Disease Biology, Liverpool School of Tropical Medicine, Liverpool, United Kingdom; 3 Mathematical Modelling Group, Oxford University Clinical Research Unit (OUCRU), Ho Chi Minh City, Vietnam; 4 Centre for Tropical Medicine and Global Health, Nuffield Department of Medicine, University of Oxford, United Kingdom; 5 Centre Suisse de Recherches Scientifiques, Abidjan, Cote d’Ivoire; 6 Ministère de la Santé, Direction de la Promotion de la Santé, Ouagadougou, Burkina Faso; 7 Ministère de la Santé, Direction de la Prévention de la Santé de la Population, Programme National de lute contre les Maladies Tropicales Négligées, Ouagadougou, Burkina Faso; 8 National Infection Service, Public Health England Colindale, London, United Kingdom; 9 Institute of infection, Veterinary and Ecological Sciences, University of Liverpool, Liverpool, United Kingdom; 10 School of Community Health Sciences, Njala University, Bo Campus, Bo, Sierra Leone; Fundação de Medicina Tropical Doutor Heitor Vieira Dourado, BRAZIL

## Abstract

**Background:**

Snakebite has become better recognized as a significant cause of death and disability in Sub-Saharan Africa, but the health economic consequences to victims and health infrastructures serving them remain poorly understood. This information gap is important as it provides an evidence-base guiding national and international health policy decision making on the most cost-effective interventions to better manage snakebite. Here, we assessed hospital-based data to estimate the health economic burden of snakebite in three regions of Burkina Faso (Centre-Ouest, Hauts Bassins and Sud-Ouest).

**Methodology:**

Primary data of snakebite victims admitted to regional and district health facilities (eg, number of admissions, mortality, hospital bed days occupied) was collected in three regions over 17 months in 2013/14. The health burden of snakebite was assessed using Disability-Adjusted Life Years (DALYs) calculations based upon hospitalisation, mortality and disability data from admitted patients amongst other inputs from secondary sources (eg, populations, life-expectancy and age-weighting constants). An activity-based costing approach to determine the direct cost of snake envenoming included unit costs of clinical staff wages, antivenom, supportive care and equipment extracted from context-relevant literature.

**Findings:**

The 10,165 snakebite victims admitted to hospital occupied 28,164 hospital bed days over 17 months. The annual rate of hospitalisation and mortality of admitted snakebite victims was 173 and 1.39/100,000 population, respectively. The estimated annual (i) DALYs lost was 2,153 (0.52/1,000) and (ii) cost to hospitals was USD 506,413 (USD 49/hospitalisation) in these three regions of Burkina Faso. These costs appeared to be influenced by the number of patients receiving antivenom (10.90% in total) in each area (highest in Sud-Ouest) and the type of health facility.

**Conclusion:**

The economic burden of snake envenoming is primarily shouldered by the rural health centres closest to snakebite victims–facilities that are typically least well equipped or resourced to manage this burden. Our study highlights the need for more research in other regions/countries to demonstrate the burden of snakebite and the socioeconomic benefits of its management. This evidence can guide the most cost-effective intervention from government and development partners to meet the snakebite-management needs of rural communities and their health centres.

## Author summary

The World Health Organisation has established a strategy to halve snakebite mortality and morbidity by 2030. Achieving this ambitious target within a decade will require substantial investment from governments of countries most affected by snakebite. The burden of snakebite however, is typically greatest in low-middle income countries with already limited health budgets. Acquiring government support to prioritise snakebite over other prevailing diseases will require evidence of the scale, causes, precise geographies and health economic impacts of snakebite. While the snakebite research community has progressed the delivery of some of these evidence types, it has been weak at providing evidence of the health economic burden of snakebite. Our hospital-based study identifies the health (Disability-Adjusted Life Years) and financial burdens of snakebite to three districts of Burkina Faso. We argue that funding of more health economic research, performed at greater depth and that includes cost-effectiveness of snakebite treatment and other remedial interventions is arguably the most effective tool to advocate for the policy support and investment required of national and international health agencies to deliver WHO’s laudable 2030 target for snakebite.

## Introduction

The World Health Organization recently launched a strategy to halve the global annual snakebite deaths (83,000–138,00) and disabilities (400,000) by 2030 [1,2]. Snakebite mortality and morbidity burdens are the highest in tropical countries, particularly in Asia and Africa [3,4]. Snakebite can be considered a disease of rural poverty as demonstrated by the statistically significant linear relationships between snakebite mortality rates and indices of poverty such as the human development index and annual government expenditure on health [5]. Snakebite induced fatalities, morbidities and consequent loss of economic productivity add to the burden of rural tropical populations that also suffer from high levels of other diseases of poverty, which can be attributed to under-resourced, poorly accessible health services and insufficient economic well-being.

Remedial intervention by health agencies and governments, especially in regions with competing health priorities and limited resources, requires these agencies to have evidence of the scale, causes, precise geographies and health economic impacts of snakebite [6,7]. In terms of cause in sub-Saharan Africa, the recent Médecins Sans Frontières publication [8] identified the need for urgent investment in the production of safe and effective antivenom for sub-Saharan Africa, and the need for clinical trials. A meta-analysis of the literature on snakebite incidence and mortality in sub-Saharan Africa [9] provided a significant advance in terms of scale and geographies of snakebite: estimating an annual mortality of 7,331 rural snakebite victims and between 5,908–14,614 amputations performed on snakebite survivors. Habib et al 2015 expressed this literature information, from 16 studies in West Africa, in terms of Disability Adjusted Life Years (DALYs) and estimated that the snakebite burden across West Africa to be 319,874 DALYs (95% Confidence Interval 245,375–402,654) [10]. The authors highlighted that whilst the snakebite DALY estimate equates to that of other neglected diseases including Buruli Ulcer, Echinococcosis, Leprosy, Trachoma, Yaws and Yellow Fever, the national, regional and international investment in managing snakebite falls far short of that devoted to these other diseases [10]. Evidence of the health economic impact of snakebite and its treatment would help advocate for change to balance this disparity. Estimating the cost of managing snakebite envenoming at local hospital level is effective in highlighting gaps and informing decision making by governments and other relevant stakeholders as to the most cost-effective allocation of resources [11], evidence that is greatly lacking in sub-Saharan Africa.

This hospital-based study presents information on the health and economic burden of snakebite in three regions of Burkina Faso. This analysis would ideally have been conducted upon detailed epidemiological data collected from both community- and hospital-based surveys of snakebite victims. However, these types of surveys are expensive and thus rarely performed (particularly in a domain like snakebite that has been under-funded and under-researched for decades) and were beyond our resources. Instead, to achieve this objective, we teamed up with better-funded colleagues undertaking a Mass Drug Administration (MDA) program for lymphatic filariasis in three regions in southern Burkina Faso to cost-efficiently collect data from snakebite victims admitted to hospitals participating in this MDA program.

## Methods

### Ethics statement

The primary data used in this study did not include information on individual patients. Data were limited to routine information that is available to technical staff of the Ministry of Health for reports and publications that are authorised by the Ministry through the National Neglected Diseases Control Program. A formal ethical approval was not required for this analysis of ministry of health data because ministry of health officials coordinating the National NTD Control Programme collected the data for this study.

### Study setting

**Fig 1:**
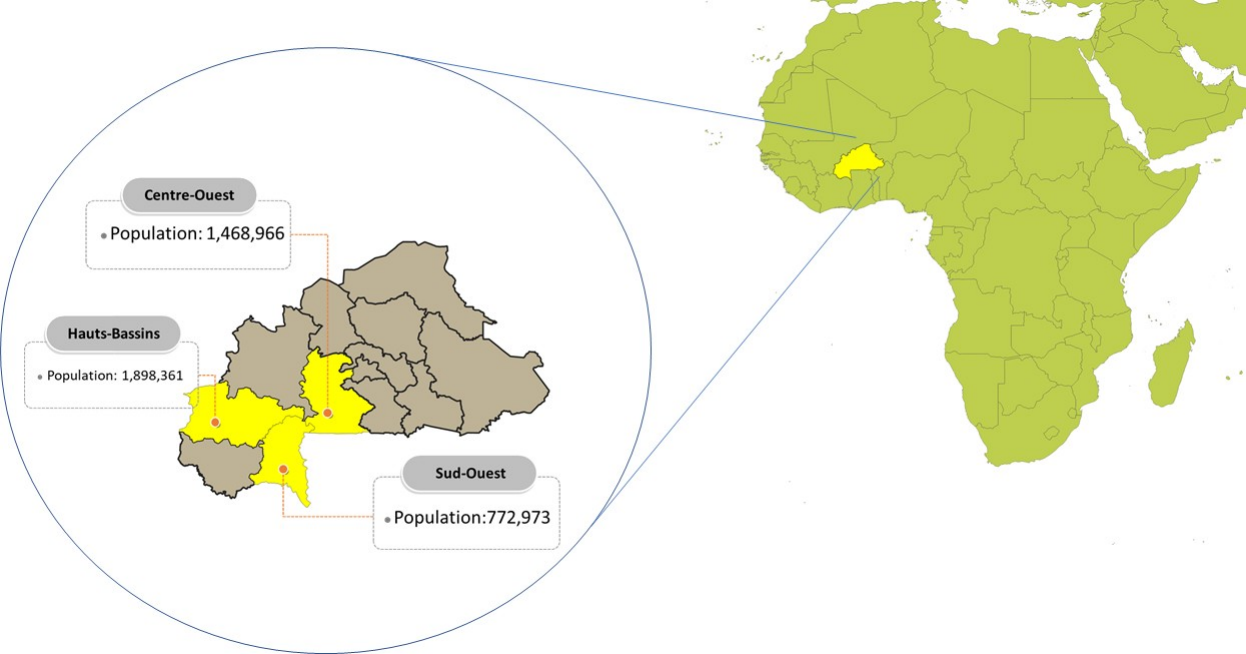
The three regions of Burkina Faso included in this study. This map was compiled using base-layer maps from “https://www.naturalearthdata.com/downloads/10m-cultural-vectors/10m-admin-0-countries” and “https://datacatalog.worldbank.org/dataset/burkina-faso-administrative-boundaries-2017”.

This study was conducted in three regions (Centre-Ouest, Hauts Bassins and Sud-Ouest; Fig 1) of Burkina Faso with a high burden of snakebite [12]. The total population of these three states was 4,140,300 in 2014 [13]. More socio-economic and health systems indicators (e.g. total population, poverty headcount, density of physicians and nurses per 1,000 population) on Burkina Faso are provided in S1 Table.

### Data collection

Both primary and secondary data were employed in this study. Primary data were gathered from regional and district health centres in three regions of Burkina Faso (Centre-Ouest, Sud-Ouest and Hauts Bassins) that participated in Mass Drug Administration programs for Lymphatic Filariasis. Data collection took place over 17 months from June 2013 to October 2014. Healthcare professionals were asked to prospectively complete data collection forms (S2 Table) concerning hospital admissions of snakebite victims and the services provided to them using routine information collected for reports and publications of Ministry of Health. Cost information including the average wages of doctors and nurses and the data on costs of necessary equipment and treatments were extracted from health facility and existing literature [10,14]. In Burkina Faso health, services are organized into three levels:

- Community health centers deliver basic preventive and curative primary healthcare [15]
- Regional/district hospitals represent the point of referral for primary healthcare centers and are managed by a district health management team led by a medical officer [16]
- Teaching hospitals at the central/national level which provides more specialized services

Funding of these facilities is largely from central government [17]. We included two types of health facilities in the three regions of Burkina Faso in this study namely:

- health centres (Centre de Santé et de Promotion Sociale (CSPS)/Centre Médical (CM)/Centre médical avec Antenne chirurgicale (CMA))
- regional or district hospitals (Centre Hospitalier Régional de Gaoua/CHR GAOUA and Centre Hospitalier Regional de Koudougou/CHR KDG)

We have excluded data collected from the teaching hospital (Hospitalier Universitaire Sanou Souro/CHU SS) because this was limited only to out-patient consultation.

### Health burden

We calculated the health burden of snakebite in terms of Disability Adjusted Life Years (DALYs) using a WHO-provided template spreadsheet [18]. We analysed hospital data to determine the annual number of admitted snakebite patients and weighted their severity using a disability weighting of 1 for the death of a snakebite patient and 0.163 for a snakebite victim suffering prolonged disability (equating to a loss of 83.7% of good health). In the absence of accepted snakebite weightings, these DALY figures were adapted from ‘poisoning’ [19] as deployed in previous snakebite DALY studies in Sri Lanka and Nigeria [10,20]. After weighting, the annual number of life years lost due to premature mortality (YLL) and years lived with disability (YLD) were determined, and the number of DALYs calculated as a sum of these: DALYs = YLL+YLD

Following formulas were used to estimate YLL and YLD in this template,

$$YLL=(K{Ce}^{ra})/{\left(r+\beta \right)}^{2}[e^{-(r+\beta )(L+a)}[–(r+\beta )(L+a)–1]-e^{-(r+\beta )a}[–(r+\beta )a–1]]+(1–K)/r(1–e^{-rL})$$ (1)

$$\mathrm{YLD}=\mathrm{DW}\{{\mathrm{KCe}}^{\mathrm{ra}}/(r+\beta {)}^{2}[e{-}^{\left(r+\beta )(L+a\right)}[–(r+\beta )(L+a)–1]–e{-}^{(r+\beta )a}[–(r+\beta )a–1]]+(1–K)/r(1–e^{-\mathrm{rL}})\}$$ (2)

Where, a = age of death/disability (years); r = discount rate (r = 3%); β = age weighting constant (β = 0.04); K = age-weighting modulation constant (K = 1); C = adjustment constant for age-weights (0.1658); L = standard life expectancy at age of death/disability (years); DW = disability weight (DW = 0.163, metastasis stage).

We inputted populations, the incidence of snakebite envenoming and mortality, life expectancy, and duration of illness by age group in the DALY estimation template. We also inserted disability weight (0.163) for ‘poisoning’ from the global burden of disease study [19,20,21]–the closest available metric to snake envenoming. Standard life expectancy by age for Burkina Faso was obtained from the life table of Global Health Observatory data repository 2017 [22].

### Economic burden

The direct economic burden of snakebite treatment to the health service providers of the three Burkina Faso regions was calculated using an activity-based costing (ABC) approach. In this approach, the services required to effectively manage the admitted snakebite patients were identified and unit costs were estimated for each service [23,24] and included all the costs incurred by the health facility for delivering the services to the patient [25]–see Table 1.

**Table 1:** Economic burden estimation of hospital services to snakebite patients.

Activity/Cost items	Unit cost / per item cost	Cost per patient (calculation and inflation adjustment	Sources
1. Doctor	Average yearly salary in 2014 = US$16,000 Daily Salary = US$43.40 Hourly Salary = US$4.34	60 minutes spent per hospitalised patient per day = $4.34	Health facility
2. Nurse	Average yearly Salary in 2014 = US$2,200 Daily Salary = US$6.02 Hourly Salary = US$0.60	1 hour spent with envenomed patient per day = $0.60 x 1	Health facility
3. Hospital bed cost	Average cost of hospital bed per day in 2014 US$4.42		Health facility
4. Diagnostic test (20 WBCT*)	Included cost for 5ml syringe, small clean dry test-tube and accessories (local sterilization with methylated spirit and tourniquet which are used for the WBCT test Cost per test = $0.31	10 tests in 7 days for a patient bitten by carpet viper (pCV = 85%) and 1 test for a non-carpet viper bite (15%) Average number of tests per patient = 0.85*10+0.15*1 = 8.65 Cost of test per patient = $0.3125×8.65 = $2.70	[26] [27] [28]
5. Antivenom cost	1 vial = $78 (average cost per dose in 2014)	On average 2.53 doses of antivenom was used per patient = $ 197.34 Gampini et. al estimated in 2014, 991 snakebite patients (4% of 24,779) received in total 2,509 doses of antivenom. Therefore on average 2.53 doses (2,509/991) were used per patient.	[12]
6. Supportive care	Analgesia (paracetamol, tramadol), blood transfusion, intravenous rehydration fluids and surgery especially debridement $18.75 per patient	$18.75 per patient	[27]
7. Treatment of early adverse reactions	Use of adrenaline injections, antihistamines (chlopheniramine or promethazine injections) and or steroids (hydrocortisone injection): $1.88 per patient	The proportion of carpet viper patient receive early adverse reaction treatment = 0.19 The proportion of non-carpet viper patient receive early adverse reaction treatment = 0.26 Proportion of snakebite victim receive early adverse reaction treatment = 0.19*0.66+0.34*0.26 = 0.21	[27]

*20 Minute Whole Blood Clotting Test (WBCT)

The average salary costs of the doctors and nurses was obtained from McCoy *et al*. (2008) and approximations of treatment costs and consumables was described by Habib et al. (2015) [10,14] from studies performed in Nigerian clinical settings that approximate to those in Burkina Faso. Ideally, this information would have been collected by the data-entry form but the unfunded nature of our study dictated a limited scale of work we could ask of our collaborators.

The cost of snakebite treatment to the health infrastructure was calculated using the data collected from the hospitals. The average daily cost for treating a snakebite patient was multiplied by the total number of hospital bed days reported from each health district. The cost per hospital stay was multiplied by the total number of admissions within each health district. The total cost of antivenom used by each health district was then determined by multiplying the average cost of treating one patient with antivenom with the total number of patients receiving antivenom in each health district. The sum of the estimated cost for each activity was used to estimate the cost of snakebite to each health district in Burkina Faso throughout the 17 months study period. The figure was then adjusted to give the annual cost to each district and to each regional health centre. Finally, an average cost per bed day was determined for each location by dividing the total costs to each district throughout the 17 months study period by the total number of bed days occupied by envenomed patients in that district. We used 2014 as the base year for estimating cost and exacting the US$ value. One US$ was equivalent to 493.76 CFA Franc in 2014 [14].

In order to test for the robustness of the two main outcomes (i.e. the total hospitalisation cost and the hospitalization cost per patient); two univariate sensitivity analyses were performed. Each input parameter was individually varied by 25%.

## Results

### Hospitalisation, death and disability of snakebite patients

The annual rate of snakebite patient hospital admissions was 173 per 100,000 population. The highest admission rate was in Sud-Ouest (342.5 per 100,000) followed by Centre-Ouest (164.7 per 100,000) and lowest in Hauts Bassins (111.0 per 100,000). The annual death rate was 1.4 per 100,000 population for the three regions and highest (3.47/100,000) in the Sud-Ouest region (Table 2).

**Table 2:** Hospitalisation and death of snakebite victims over 17 months period between June 2013 and October 2014.

Region		Centre-Ouest	Hauts Bassins	Sud-Ouest	Total
Population*		1,468,966	1,898,361	772,973	4,140,300
**Snakebite related hospitalization**	Number of patients hospitalized in 17 months	3,428	2,986	3,751	10,165
	Annual rate of snakebite related hospitalization per 100,000	164.70	111.0	342.5	173.3
	(95% CI)	(158.30–171.40)	(106.40–115.90)	(329.70–355.90)	(169.30–77.40)
**Death due to snakebite**	Total death 17 months	20	24	38	82
	Annual death rate per 100,000	0.96	0.89	3.47	1.39
	(95% CI)	(0.52–1.60)	(0.52–1.43)	(2.30–5.08)	(1.06–1.81)

*Source: Institut national de la statistique et de la démographie 2014 [13]

**Fig 2:**
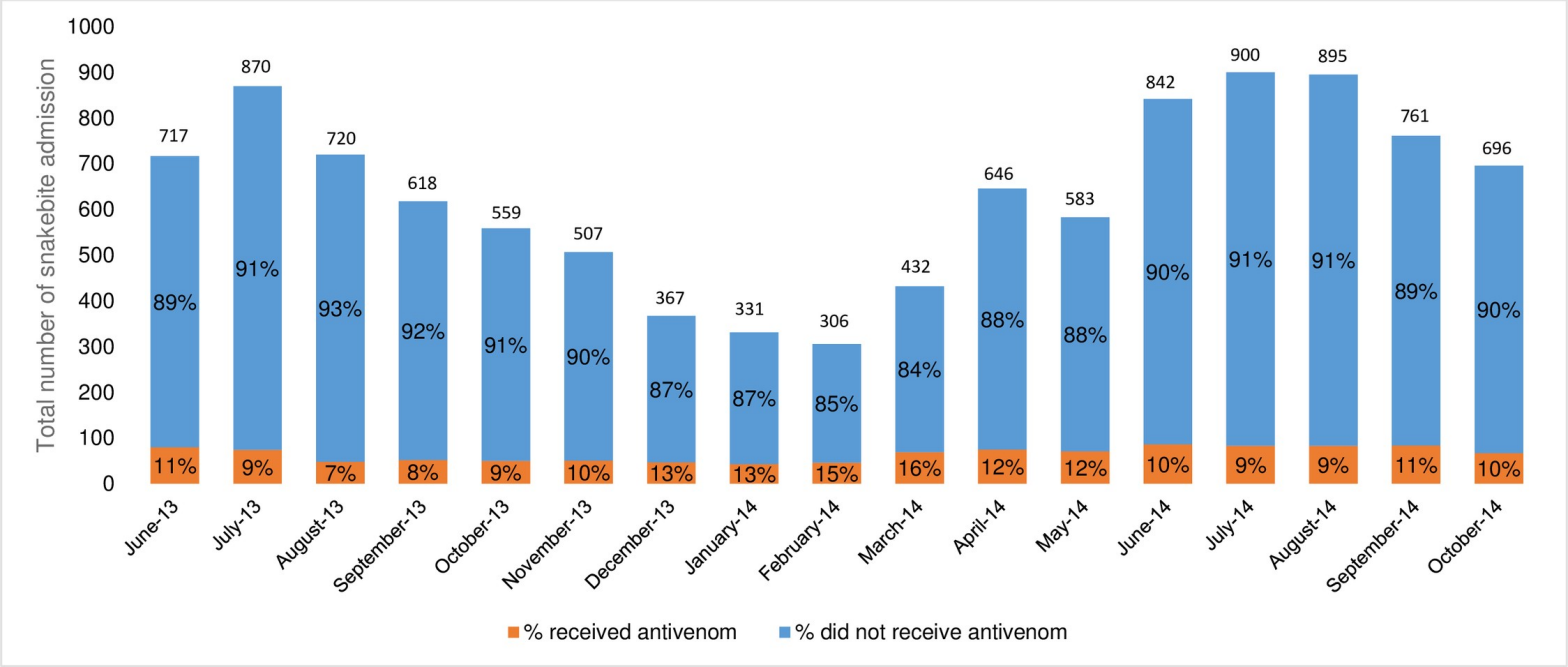
Number of hospitalised snakebite patients in the three regions studied and the percentage of patients that did, and did not receive antivenom treatment.

Fig 2 illustrates the seasonal nature of snakebite in Burkina Faso, with hospital admissions over the 17 months period peaking during the cooler and wetter months of May/June to August/September and dropping during the driest months of December to January–typical for West Africa. The rain season is the period of peak agricultural activity and thus the period when subsistent famers are at greatest risk of snakebite [5]. On average 11% of the hospitalized snakebite victims received antivenom.

### Hospitalisation, death and disability of snakebite patients

Based upon the data available to us, snakebite accounts for the annual loss of 2,153 DALYs (0.52 per 1,000) in these three regions of Burkina Faso (Table 3). The highest DALYs lost was in the Sud-Ouest region followed by Hauts Bassins and Centre-Ouest. In total DALYs lost, the share of Years Life Lost (60%) was higher than the share of Years Lived with Disability (40%).

**Table 3:** Annual disease burden of snake envenoming in three regions of Burkina Faso (hospitalised patients only).

Region	Years Lived with Disability (YLD)	Years of Life Lost (YLL)	Disability-adjusted life year (DALY)	DALY per 1,000
Centre-Ouest	257	341	598	0.41
Hauts Bassins	221	409	630	0.33
Sud-Ouest	277	648	925	1.20
**Total**	**755**	**1,398**	**2,153**	**0.52**

**DALY estimation parameters:** Mean age at bite = 25–29 years; Remaining life expectancy = 43 years; [28] Disability weight = 0.163 [20]; Discount rate = 3%; For YLD estimation, 3.2% patients were considered to suffer long term disability with an average of 13.4 years [29]

A total of 10,165 snakebite patients were admitted to the collaborating hospitals and occupied a total of 28,164 hospital bed days over the 17 months study period (Table 4). The average length of stay in hospital was 2.6 days. A total of 1,109 patients received antivenom treatment and 10 patients required surgery to manage venom-induced local tissue damage. The highest number of hospitalisation services were delivered by CSPS/CM/CMA facilities. The average length of stay was the highest in CHR GAOUA facilities (3.9 days) and followed by CHR KDG (3.0 days) and CSPS/CM/CMA (2.5 days).

**Table 4:** Health service utilisation for snake envenoming by region and type of health facility (17 months period).

Region/health facility types	Number of hospital admission	Total hospital days	Length of stay		Antivenom received	Surgery (e.g. debridement or amputation)
			Mean	95% CI		
**Region**						
Centre-Ouest	3,428	8,311	2.4	(2.3–2.6)	62	1
Hauts Bassins	2,986	6,610	2.0	(1.9–2.2)	118	2
Sud-Ouest	3,751	13,243	3.5	(3.3–3.8)	929	7
**Total**	**10,165**	**28,164**	**2.6**	**(2.5–2.7)**	**1,109**	**10**
**Types of health facility**						
CHR GAOUA^a^	399	1,571	3.9	(3.7–4.1)	346	7
CHR KDG^b^	153	471	3.0	(2.6–3.4)	43	-
CSPS/CM/CMA^c^	10198	26,122	2.5	(2.4–2.6)	720	3

^a^CHR GAOUA: Centre hospitalier régional de Gaoua (Regional/district hospital)

^b^CHR KDG: Centre hospitalier regional de Koudougou (Regional/district hospital)

^c^CSPS/CM/CMA: Centre de Santé et de Promotion Sociale/Centre Médical/Centre médical avec Antenne chirurgicale (Health centers)

### Antivenom treatment of snakebite victims

**Fig 3:**
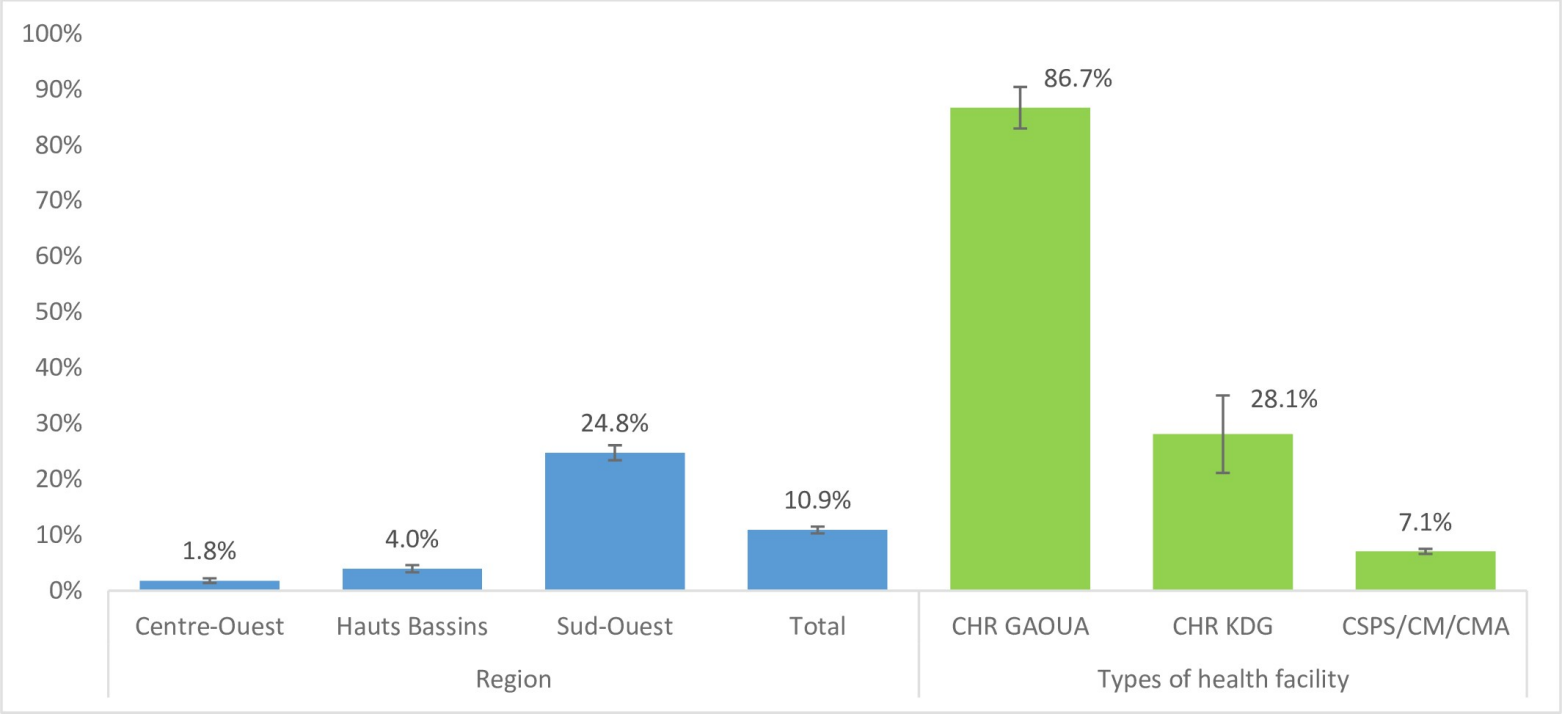
Percentage of hospitalised snakebite patients that received antivenom treatment—by region (blue bars) and facility type (green bars).

A total of 10.9% snakebite victims received antivenom treatment (Fig 3). More patients admitted to hospitals in the Sud-Ouest region received such treatment (24.8%) than in the other regions. Most (86.7%) of the treated patients received antivenom from CHR GAOUA hospitals.

**Fig 4:**
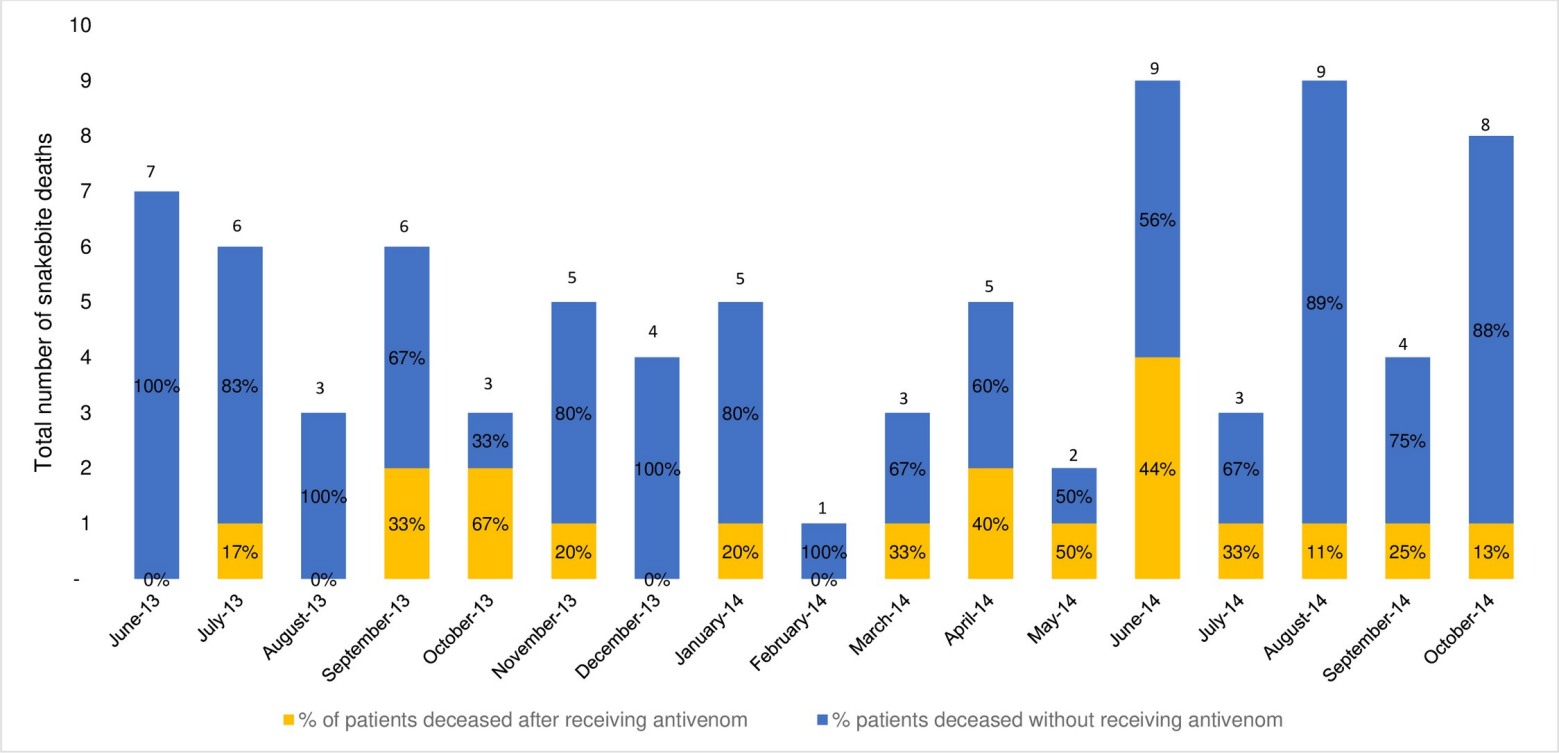
Number of hospitalised snakebite patient deaths and percentage deaths in patients who did, and did not receive antivenom treatment.

The monthly mortality of hospitalised snakebite patients and the percentage of patients that died after receiving antivenom treatment and without treatment is presented in Fig 4. Patients not receiving antivenom exhibited a higher share of total deaths than antivenom treated patients.

### Economic burden

The estimated cost per bed day occupied by envenomed patients varied substantially between locations and facility types (Table 5). The annual costs of hospital services to snakebite patients was USD 506,413. The per-patient cost for hospitalisation was USD 49. This was the highest in CHR GAOUA (USD 423) facility followed by CSPS/CM/CMA (USD 42). Sud-Ouest has a higher per patient hospitalisation cost compared to the Hauts Bassins and Centre-Ouest regions.

**Table 5:** Annual cost (USD) of hospital management of snakebite patients by region and types of health facility.

Region/types of facility	Hospitalisation cost (USD^a^)								
	Doctor	Nurse	Hospital bed cost	Diagnostic test	Antivenom cost	Supportive care	EARM^b^ cost	Total cost	Per patient cost
**Region**									
Centre-Ouest	25,461	3,532	25,930	6,552	8,637	45,503	973	116,588	37
Hauts Bassins	20,250	2,809	20,623	6,787	16,437	47,131	1,008	115,045	34
Sud-Ouest	40,570	5,627	41,318	7,149	129,409	49,646	1,061	274,781	85
**Types of facility**									
CHR KDG^c^	1,443	200	1,470	292	5,990	2,025	43	11,462	68
CHR GAOUA^d^	4,813	668	4,902	760	48,197	5,281	113	64,734	161
CSPS/CM/CMA^e^	80,026	11,100	81,501	19,436	100,295	134,974	2,886	430,217	41
**Total**	86,281	11,968	87,872	20,488	154,482	142,279	3,042	506,413	49

^a^United States Dollar

^b^Early adverse reaction management

^c^Centre Hospitalier Regional de Koudougou

^d^Centre Hospitalier Régional de Gaoua; ^e^Centre de Santé et de Promotion Sociale (CSPS)/Centre Médical (CM)/Centre médical avec Antenne chirurgicale (CMA)

**Fig 5:**
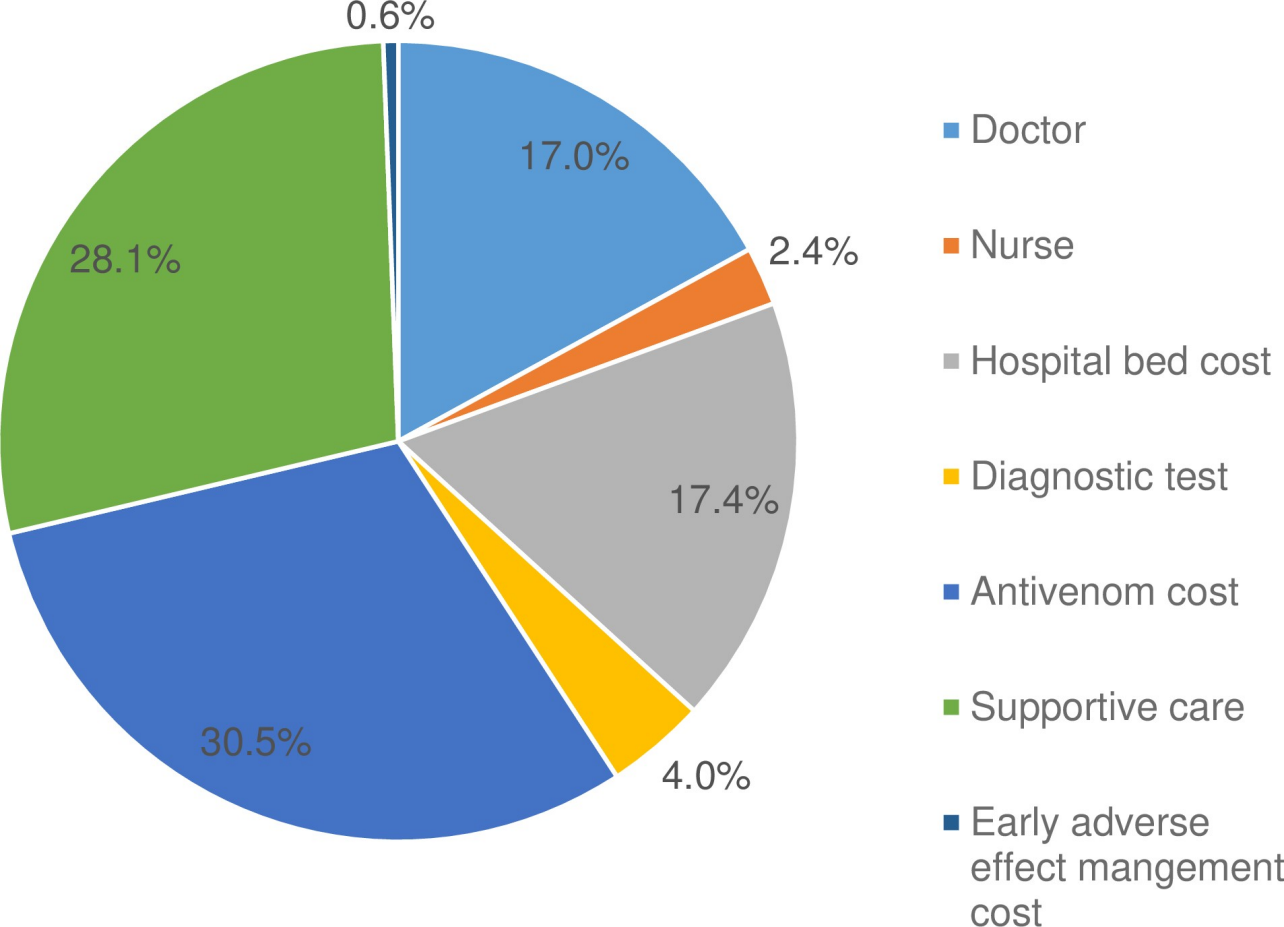
The percentage share of total hospitalisation cost by item.

Fig 5 illustrates that of all the different hospitalisation costs, the highest was incurred by antivenom use (30.5%) followed closely by supportive care (28.1%) and lower costs for hospital beds (17.4%) and doctors’ visits (17.0%).

**Fig 6:**
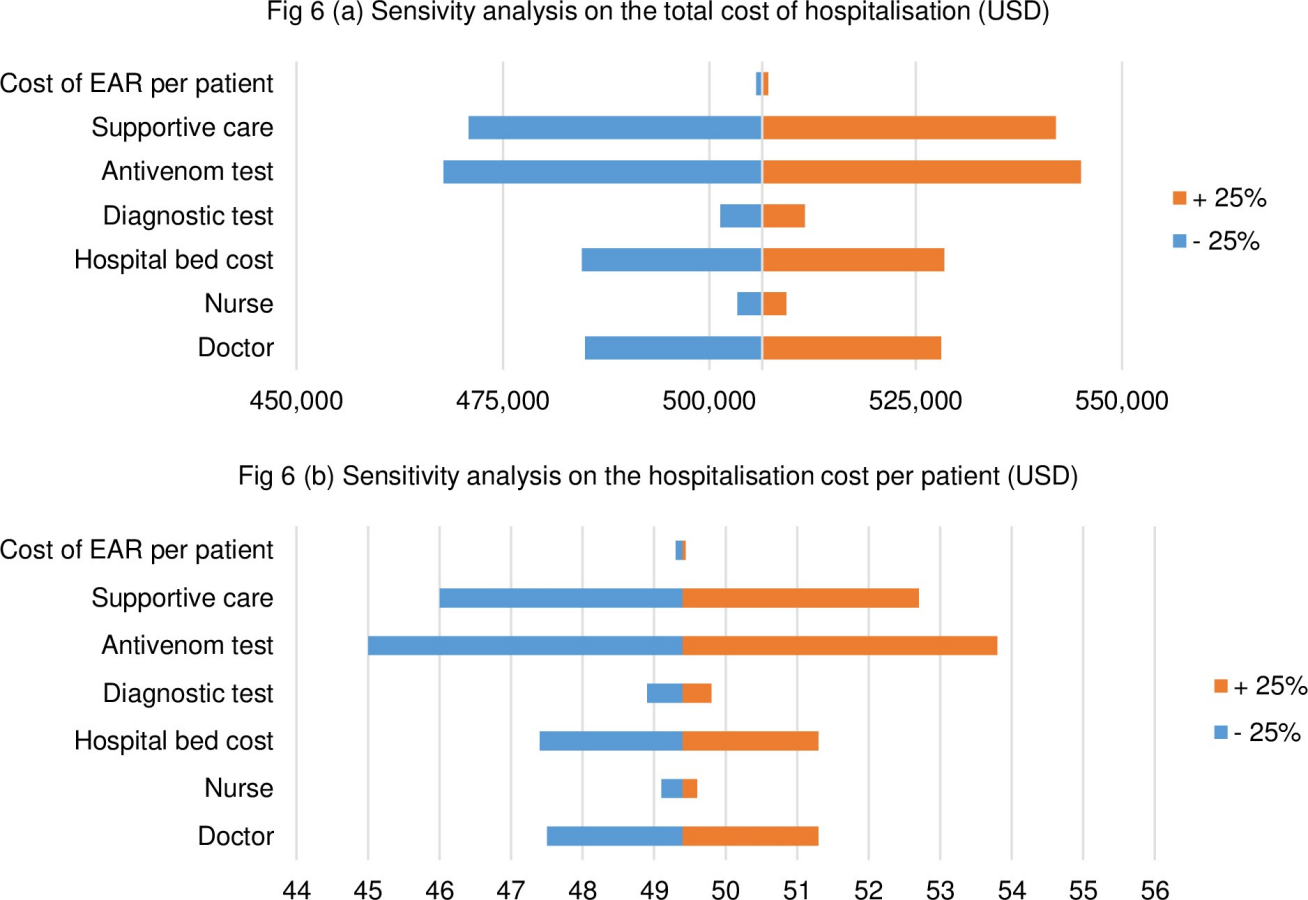
One way sensitivity analysis on the total hospitalization cost and the hospitalization cost per patient.

The results of the one-way sensitivity analysis are reported in tornado diagrams in Fig 6 below. Hospitalisation costs are sensitive to variations in cost of the antivenom test and in the spending on supportive care. The cost of EAR per patient does not have a strong effect on the outcome.

## Discussion

The aforementioned 2019 WHO strategy to halve global snakebite mortality and morbidity by 2030 was mandated (WHA71.5) by the 2018 World Health Assembly [30] resolution on snakebite envenoming. This followed the 2017 decision by the WHO to add snakebite as one of its priority Neglected Tropical Disease [31]. That WHA resolution was supported by 31 countries and based upon admittedly weak incidence, mortality and morbidity figures [30]. No mention was made of conventional health economic burden/impact statements, presumably because these were unavailable for snakebite. Providing evidence of the health economic impact of snakebite will assuredly help retain/strengthen support of these and other governments, and of international health agencies and donors to guide their health intervention policies, which, in turn, will help WHO achieve its snakebite strategy objectives.

Burkina Faso is a low-income Sahelian country with 40.1% of the population living below the national poverty line, whose economy is dependent upon agriculture that employs 80% of the working population [32]. The circumstances of these remote, predominantly subsistent-farming, impoverished communities place them at particularly high snakebite risk [5–7] and are precisely the type of communities that the WHO hope will benefit most from their new strategy. In that context, the present study was undertaken to deliver new information on the health and economic burden of snakebite to rural hospitals in three southern regions of Burkina Faso.

Over the 17 months of this study in Centre-Ouest, Hauts Bassins and Sud-Ouest regions of Bukina Faso, 10,165 snakebite patients were admitted to hospital (173.3/100,000; Table 2), particularly during the wetter May-September periods, where they consumed 28,164 hospital bed days. In terms of medical and geographic impact, the Sud-Ouest region shouldered the greatest snakebite admission burden (342 admitted patients/100,000 population) and costs of antivenom administration (Fig 3). The majority (24.8%) of patients were admitted to CHR GAOUA hospitals and it was these facilities that administered the vast majority (86.7%) of antivenom treatments. This is because CHR hospitals manage more severe snakebite patients who are referred from CSPS/CM/CMA facilities. Furthermore, the inhabitants of the south-west (Gaoua) region are more rural than communities in the other two regions.

Of the total 10,165 admitted snakebite patients, 82 patients did not survive (0.8%; Table 2) and 1,109 were treated with antivenom (10.9%). Antivenom administration was clearly associated with clinical benefit since the percentage of monthly deaths amongst treated patients was substantially lower (ranging from 11–67%) than deaths of untreated patients (ranging from 33–100%; Fig 4). Our study was unable to determine whether the deaths of the untreated patients was attributable to a lack of antivenom, to patients arriving with such severe pathologies that antivenom administration was too late to prevent death, or to other causes. Notably only 10 patients (0.09% of all admitted patients and 0.9% of treated patients) received debridement or amputation surgery to correct venom-induced tissue damage–a figure that questions the accuracy of the widely-accepted estimate that snakebite induced morbidity is 3–4 fold higher than mortality rates. Despite extensive seasonal variation in monthly patient admission (Fig 2) and mortality (Fig 4) rates, the rate of antivenom treatment was more constant (average of 11% patients, Fig 2). The important 2016 Burkina Faso-wide, retrospective snakebite study by Gampini et al examined centralised hospital records from 2010–2014 and determined a national snakebite incidence rate of 136/100,000, a 1.2% mortality rate and also identified the Sud-Ouest region as being at highest snakebite risk [12]. This study provided particularly valuable information on the annually changing availability and cost of the antivenoms in Burkina Faso (FavAfrique; Sanofi, France and EchiTAb-Plus-ICP; Instituto Clodomiro Picado, Costa Rica).

In terms of snakebite burden estimates, our analysis of the data from these 3 regions of Burkina Faso yielded an annual estimated 2,153 DALYS/year, equating to 0.52/1,000 population. The total estimated hospital cost of managing one snakebite patient was approximated at USD 49. These DALY figures are nearly identical to the 0.5 DALYs/1,000 determined for snakebite patients in Sri Lanka in 2013 [20], where cost of treatment (USD 128.8) was however three-fold higher than in Burkina Faso. Two other sub-Saharan Africa studies also estimated hospital costs of treating snakebite patients. In neighbouring NE Nigeria, where the *Echis ocellatus* saw-scaled viper also dominates snakebite incidence and mortality, treatment costs with EchiTAb-Plus-ICP and the saw-scaled viper-specific antivenom, EchiTAbG (MicroPharm, Wales) were estimated at USD 216.25 [10]. In KwaZulu Natal where the dominant biting species, *Naja mossambica* spitting cobra causes extensive local tissue, hospital treatment costs with the SAIMR polyvalent antivenom (South Africa Vaccine Producer, South Africa) were identified as ranging between USD 1,156–2,827 [33]–substantially higher than in Burkina Faso. In the context of other prevailing tropical diseases, it is instructive to note that the estimated costs/patient of treating uncomplicated and complicated Malaria were USD 5.85 and USD 30.26 respectively [34], presumably reflecting the difference between out- and in-patient treatment costs and that hospital management of a snakebite patient is more expensive than a complicated malaria patient.

We determined that the overall annual cost to hospitals of snakebite treatment in three states of Burkina Faso was USD 506,413, with the burden being greatest in regional health centres (e.g. CSPS/CM/CMA). The variation in cost between locations and facility types appeared to be influenced by the number of patients receiving antivenom treatment in each area (e.g. Sud-Ouest) and health facility (e.g. CHR GAOUA). Approximately 11% of hospital admissions were treated with antivenom over the study period–this is more than double the Gampini et al figure of 3.9% patients treated in the 4 years prior to our study [12]. This discrepancy might reflect annually changing volumes of antivenom supplies, regional variation in antivenom availability (we studied only 3 regions) and data/or collected from hospitals (ourselves) versus from central records.

A landmark study examining the burden of snakebite across West Africa [10] determined that hospital treatment of other more recognized NTDs (eg, trachoma, onchocerciais, lymphatic filariasis) received between USD 3.30 and 146.96 per DALY averted. In contrast, no such funding existed then for snakebite. The authors emphasized that antivenom treatment of snakebite has proved highly cost-effective and, with current costs per DALY averted ranging between USD 56.88 to USD 99.61 (dependent on discounts made), represents a valuable allocation of health resources [10]. A separate study assessed that lowering the price of antivenom to USD 40/treatment would incur a USD 10 per DALY averted–a superior cost-effectiveness compared to other NTDs previously described [35]. This is heavily supported by the sensitivity analyses (Fig 6), which show that the cost of antivenom (as well as the cost of supportive care) influence heavily the total cost of hospitalisation, which in turn influences the cost-effectiveness of snakebite interventions.

In Burkina Faso, the total DALYs per 1,000 population was 63.31. Therefore, the share of DALYs burden related to snakebite was 0.82% of total. According to The World Bank, the per capita health expenditure in Burkina Faso was US$ 39.59 in 2014. Considering the total population, the total health expenditure in the three selected regions (Centre-Ouest, Hauts Bassins and Sud-Ouest) was US$ 164 million. Thus in the selected regions, the share of snakebite hospitalization cost was 0.31% of the total health expenditure in 2014.

Health financing is a major challenge to the Burkina Faso Ministry of Health, with general government expenditure on healthcare estimated at 12.8% of total government expenditure [36]. The Burkina Faso government has provided an atypically high level of support for snakebite management: in 2010 snakebite was made a centrally notifiable event and, in 2015, the price of antivenom was subsidised to USD 3.4—less than 5% of the commercial rate [12]. Such laudable health policy changes would be expected to reduce the economic burden imposed on health centres in Burkina Faso and thereby reduce the mortality and morbidity of snakebite victims. If data was available that evidenced these medical and health economic outcomes from this national investment in antivenom, it would likely encourage adoption of similar health initiatives in other countries experiencing high snakebite hospital admissions.

Our study has provided an estimation of the health and economic burden of snakebite to rural hospitals in Burkina Faso. Several limitations can be described which should be taken into consideration when interpreting the results. Our poor funding base meant we were unable to conduct more detailed surveys, including of communities, to examine the disease burden, causes and costs. For example, we would have liked to determine, during the rain seasons, why monthly antivenom supply rates did not increase with rising snakebite patient admissions and deaths. Similarly, instead of relying upon estimates from other sources, we would have liked to conduct more detailed, primary-data surveys to more precisely determine facility-wide costs of snakebite management in each of the different tiers of the Burkina Faso health facilities and understand which brands of antivenom were delivered and how these medicine-supply decisions were made and their cost-effectiveness. Studies such as these provide very valuable information but are expensive and were beyond our means. We excluded analysing data on the total number of health facility consultations by snakebite victims (1.5m, Centre-Ouest; 1.8m, Haut Bassins; 0.91m, Sud-Ouest). The total of 4.2m snakebite victims seeking hospital care seemed extraordinarily high and our data collection tool was ill equipped (because of funding issues) to robustly investigate this figure–a loss of potentially very important data. Estimations of cases and fatalities were extracted only from hospital-based collection systems and it is likely that a large proportion of snakebite victims elected not to visit health facilities and so went unreported–a 1994 study of rural Kenyan communities determined that 68% of snakebite victims did not seek hospital care [37]. Our data collection system relied on participation by busy healthcare professionals and data were missing for several participating health facilities. We excluded facilities with missing data when calculating averages, which may have affected the results. Costs to hospitals should therefore only be considered as estimations. Data from only three states of one country was examined therefore caution must be applied when extrapolating results nationally and to other countries in sub-Saharan Africa.

The most successful outcomes of snake envenomed patients are attributable to rapid medical attention and administration of antivenom. The reality however, is that antivenom is often unavailable in remote hospitals closest to snakebite victims [5,6,12]. A 2011 study estimated that the availability of antivenom in Africa is less than 2.5% of the need, because of prolonged under-investment in antivenom manufacture, inconsistent antivenom demand and improper usage [38]. When antivenoms are available, a great many, perhaps the majority, of snakebite patients are unable to afford remarkably expensive antivenom treatments. A 2017 study conducted in Kenya [39] reported that antivenom purchase costs to hospitals ranged from USD 48 to 315/vial—costs that should be at least tripled given the clinical need to administer 2–5+ vials for an effective treatment. The antivenom dose is dictated by the snake species responsible, the amount of venom injected and the efficacy of the antivenom used [40]. Snakebite treatment can require additional medical interventions, including assisted ventilation to prevent respiratory collapse of paralysed snakebite victims, antibiotics to prevent infection of the bite wound and a range of other drugs to manage antivenom-induced adverse effects and to prevent renal damage and infarction [41,42]. These, together with the need for surgical intervention to manage venom-induced tissue damage and necrosis, add to the complexity of snakebite management and to the costs imposed upon under-resourced rural hospitals [5,9]. The cost of diagnostic test, supportive care and treatment of early adverse reactions were extracted from a Nigerian study due to the unavailability of country-specific estimates [27]. The unit cost estimate can differ between these two countries, which will influence the total cost estimate. However, this effect will not be very high as a major share of the total cost (67.3%) were sourced from the Burkina Faso settings (Fig 5).

Healthcare in remote rural communities is poorly accessible and often inadequately equipped to effectively manage snakebite. Regional and district level hospitals often lack the necessary financial resources, clinical expertise, laboratory instruments and pharmaceutical supplies to effectively deliver treatments. Reported shortcomings in medical training and textbooks on management of snakebite in Asian countries [43] will surely apply to other countries with lesser financial resources. Given estimates that in some rural West African hospitals up to 10% of hospital beds can be occupied by snakebite patients, especially in the rain seasons [27], it seems an imperative to equip these rural hospitals with the resources, staff and medicines they need most–and especially so because of the economic importance of subsistence agriculture in these communities. It needs to be acknowledged that health budgets of governments, international health agencies and donors are finite and carefully prioritised. It is therefore incumbent upon snakebite stakeholders to provide evidence from medical/health economic research studies to guide how and where health agencies should allocate their resources for maximum health and economic return.

Geopolitical solutions and funds to implement them are now sought to ensure that tropical snakebite patients have access to effective healthcare. It is essential that adequately funded studies are conducted which help to present information to both tropical governments and international health agencies that identify the scale, location and cost of the problem and help identify the most cost- and medically-effective interventions. The present study meets that remit to a limited (because of its inadequate funding) extent but nevertheless describes the cost of snakebite management to rural African healthcare systems, and illustrates that snakebite poses a substantial medical and economic strain on the healthcare infrastructure serving the poorest rural communities. We hope this study describes the value of, and underscores the need for more health economic research so that evidence gathers to help guide allocation of resources that meets the clinical, medicinal and staffing needs of rural tropical health facilities to effectively manage snakebite.

## Supporting information

S1 Table Socio-economic and health system indicators of Burkina Faso. (DOCX)

S2 Table Data collection form. (DOCX)
