# Supplementary material for: Health and economic burden estimates of snakebite management upon health facilities in three regions of southern Burkina Faso
Source: PLoS Negl Trop Dis. 2021 Jun 21;15(6):e0009464. doi: 10.1371/journal.pntd.0009464 (PMC8248599; doi:10.1371/journal.pntd.0009464)
Supplement: S1 Table — (DOCX) [file pntd.0009464.s001.docx]

S1 Table. Socio-economic and health system indicators of Burkina Faso

| **Indicators** | **Burkina Faso** |
| --- | --- |
| Total population (2015) [1] | 18,105,600 |
| Rural population (2014) [2] | 73% |
| Life expectancy at birth (2015) [1] | 59.9 |
| Poverty headcount ratio at international $1.25 a day (2009) [1] | 44.6 |
| Density of physicians per 1000 population (2010) [1] | 0.05 |
| Density of nurses and midwives per 1000 population (2010) [1] | 0.57 |
| Per capita health expenditure (2014) [3] | US$ 39.59 |
| Per capita GDP [4] |  |

References:

1. Global Health Observatory <http://apps.who.int/gho/data/node.cco>

2. World Development indicators. <https://data.worldbank.org/indicator/SP.RUR.TOTL.ZS?end=2014&locations=BF&start=1960>

3. World Development indicators.

https://datos.bancomundial.org/indicador/SH.XPD.CHEX.PC.CD?end=2014&locations=BF&start=2011

4. World Development indicators https://data.worldbank.org/indicator/NY.GDP.PCAP.CD?end=2014&locations=BF&start=2007
