# Supplementary figures and images for: Health and economic burden estimates of snakebite management upon health facilities in three regions of southern Burkina Faso
Source: PLoS Negl Trop Dis. 2021 Jun 21;15(6):e0009464. doi: 10.1371/journal.pntd.0009464 (PMC8248599; doi:10.1371/journal.pntd.0009464)

S2 Table. Data collection form


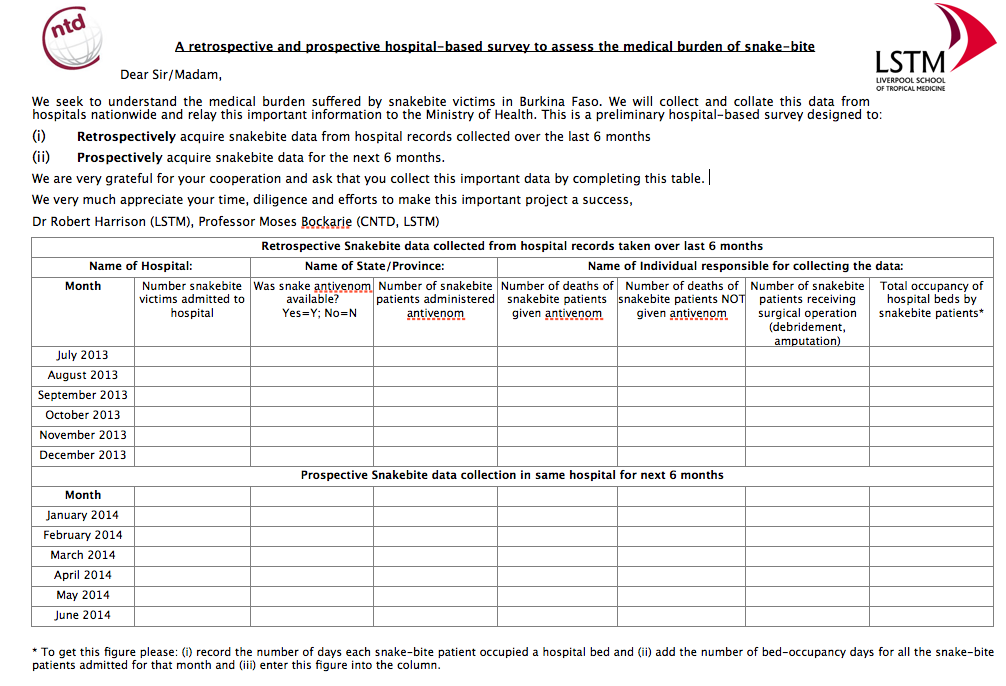

Supplement: S2 Table — (DOCX) [file pntd.0009464.s002.docx]
